# Fluopyram activates systemic resistance in soybean

**DOI:** 10.3389/fpls.2022.1020167

**Published:** 2022-10-24

**Authors:** Leonardo F. Rocha, Arjun Subedi, Mirian F. Pimentel, Jason P. Bond, Ahmad M. Fakhoury

**Affiliations:** School of Agricultural Sciences, Southern Illinois University, Carbondale, IL, United States

**Keywords:** *Glycine max*, plant-parasitic nematodes, seed treatment, SDHI fungicides, RNA-Seq, transcriptomics, induced systemic resistance, defense priming

## Abstract

The soybean cyst nematode (SCN) (*Heterodera glycines* Ichinohe) is a significant yield-limiting factor in soybean production in the Midwestern US. Several management practices are implemented to mitigate yield losses caused by SCN, including using SDHI (succinate dehydrogenase inhibitors) fungicides delivered as seed treatments. A set of studies was conducted to evaluate the effect of two seed-applied succinate dehydrogenase inhibitors (SDHI) compounds, fluopyram and pydiflumetofen, on SCN population densities, plant injury, and plant growth. Cyst counts in untreated control and pydiflumetofen treated plants were 3.44 and 3.59 times higher than fluopyram, respectively, while egg counts were 8.25 and 7.06 times higher in control and pydiflumetofen. Next-generation sequencing was later employed to identify transcriptomic shifts in gene expression profiles in fluopyram and pydiflumetofen -treated seedlings. RNA expression patterns of seed treatments clustered by sampling time (5 DAP vs. 10 DAP); therefore, downstream analysis was conducted by timepoint. At 5 DAP, 10,870 and 325 differentially expressed genes (DEG) were identified in fluopyram and pydiflumetofen, respectively. These same treatments generated 219 and 2 DEGs at 10 DAP. Multiple DEGs identified in soybean seedlings treated with fluopyram are linked to systemic resistance, suggesting a potential role of systemic resistance in the suppression of SCN by fluopyram, in addition to the known nematicidal activity. The non-target inhibition of soybean succinate dehydrogenase genes by fluopyram may be the origin of the phytotoxicity symptoms observed and potentially the source of the systemic resistance activation reported in the current study. This work helps to elucidate the mechanisms of suppression of SCN by fluopyram.

## Introduction

Plant-parasitic nematodes are major biotic factors disturbing crop production systems worldwide, limiting yield potential and profitability, exacerbating poverty, food insecurity, and malnutrition (Singh et al., 2015; Bernard et al., 2017; Coyne et al., 2018). The soybean cyst nematode (SCN) (*Heterodera glycines* Ichinohe) is the major soybean plant-parasitic nematode in the Midwestern US, with broad distribution across all main soybean production areas of the country (Niblack and Tylka, 2008; Bandara et al., 2020). Yield losses caused by SCN nationwide were estimated to be twice those caused by other combined diseases (Bradley et al., 2021). SCN management is rather challenging, as yield losses may reach up to 60% in susceptible varieties (Hershman, 2014), and often reductions of up to 30% are observed without noticing aboveground symptoms (Niblack, 2005; Mueller et al., 2016; Tylka and Marett, 2017).

A diversified toolbox of management practices is promoted to mitigate yield losses caused by SCN, including using resistant soybean varieties, crop rotation with non-hosts, weed management, seed-applied nematicides, and biological control products (Niblack and Tylka, 2008; Niblack, 2009; Mueller et al., 2016; Rocha et al., 2021a; Rocha et al., 2021b; Rocha et al., 2022). Over 90% of SCN-resistant varieties share a common resistance source, resistance genes from the plant introduction (PI) 88788. The lack of rotation among sources of resistance has led to the selection of resistant SCN populations (Niblack and Tylka, 2008).

The use of pesticides in agriculture has been increasing over the last decades, especially following the introduction of genetically modified herbicide-resistant (GMHR) crops (Mortensen et al., 2012). In soybeans, 99% of fields used herbicides, 16% insecticides, and 15% fungicides in a survey covering the 2018 cropping season (USDA-NASS, 2019). Although fungicides represent only a fraction of herbicide use in the US, from 2012-to 2014, 76% of soybean acreage used seed treatments (Hitaj et al., 2020). The use of seed treatment has been increasing since the late 1990s and early 2000s and has become a widespread tool used to manage soil-borne insects, nematodes, fungi, oomycetes, and bacteria (Munkvold, 2009; Hitaj et al., 2020). Applying chemicals *via* seed treatment may reduce active ingredients’ rates, deliver a compound directly to target organisms, and protect seedlings during germination and initial development (Roth et al., 2020).

The primary seed treatment chemistries currently used to manage SCN are abamectin, a natural compound from *Streptomyces avermitilis*, fluopyram, and pydiflumetofen (Roth et al., 2020). Fluopyram and pydiflumetofen, introduced in the last decade, are systemic succinate dehydrogenase inhibitors (SDHI) fungicides, protecting soybean seedlings against soil-borne diseases, including SCN and other plant-parasitic nematodes. Similar to fungi, the succinate dehydrogenase (SDH) complex is also the target for fluopyram and pydiflumetofen in plant-parasitic nematodes (Heiken, 2017; Schleker et al., 2022). In the mitochondrial matrix, SDH catalyzes the oxidation of succinate to fumarate, transferring electrons to ubiquinone without pumping protons across the mitochondrial inner membrane (Huang and Millar, 2013). These SDHI chemistries are classified as group 7 by the fungicide resistance action committee (FRAC (Fungicide Resistance Action Committee), 2020), acting by strongly binding to ubiquinone-binding sites (Qp) (competitive inhibition) in the succinate dehydrogenase complex, which is composed of four subunits (SdhA, SdhB, SdhC, and SdhD) (Sang et al., 2018). Fluopyram is a pyridinyl-ethyl-benzamide, while pydiflumetofen is an N-methoxy-(phenyl-ethyl)-pyrazole-carboxamide (**Figure S1**).

Fluopyram and pydiflumetofen are reported to have activity against major soybean diseases, including sudden death syndrome (*Fusarium virguliforme*) and SCN (Kandel et al., 2017; Roth et al., 2020). Both fluopyram and pydiflumetofen are SDHI fungicides with similar chemical structures, although fluopyram appears to have a broader range of activity against several fungal pathogens in field studies (Veloukas and Karaoglanidis, 2012; Watson et al., 2020), and a label recommendation for early-season Septoria brown spot (*Septoria glycines*). The fact that fluopyram, a seed-applied fungicide, has activity against a broad range of nematodes and fungal pathogens, including a soybean foliar pathogen (*S. glycines*), signals a potential connection to systemic resistance. Peng et al. (2020), using a quantitative reverse transcription PCR (RT-qPCR) approach limited to three genes (PR-1, CHI, and NCED3), reported fluopyram inducing the expression of the indicator gene coding PR-1 proteins.

Therefore, the objectives of this study were to *i*) determine the effect of fluopyram and pydiflumetofen seed treatment on plant growth and development; *ii*) explore how fluopyram and pydiflumetofen affect SCN penetration, reproduction, and fecundity; *iii*) use next-generation sequencing technologies (RNA-seq) to identify transcriptomic shifts in gene expression profiles in soybean resulting from a seed treatment with fluopyram and pydiflumetofen.

## Material and methods

### Soybean planting, SCN inoculum preparation, and infection

A greenhouse trial was conducted to assess the effect of fluopyram and pydiflumetofen applied as seed treatments on SCN infection and plant systemic response. The greenhouse experiment was conducted in a completely randomized design, with six treatments (mock, fluopyram, pydiflumetofen, SCN control, fluopyram + SCN, and pydiflumetofen + SCN) and 13 replicates (**Table 1**). The treatments without SCN inoculation (mock, fluopyram, and pydiflumetofen) were used for the RNA-seq study. In contrast, the treatments inoculated with SCN (SCN control, fluopyram + SCN, and pydiflumetofen + SCN) were utilized for SCN counts and plant growth measurements. Eight replicates were used for RNA extraction (two time points, four replicates per time point) and five for SCN counts, phenotypic analysis, and root scanning. Williams 82 (PI 518671) seeds, susceptible to SCN, were treated following the field rate recommendations for fluopyram and pydiflumetofen (**Table 1**). Seeds were mixed manually until uniform seed coverage was achieved and air-dried overnight. Non-treated seeds went through the same treatment process using autoclaved deionized water.

**Table 1:** List of treatments used for the greenhouse trials.

Assay	Treatment	Description	Seed treatment	Rate	Unit	SCN
**RNA-seq**	1	Mock	–	–	–	–
	2	Fluopyram	Fluopyram	0.15	mg ai/seed	–
	3	Pydiflumetofen	Pydiflumetofen	0.075	mg ai/seed	–
**SCN Data**	4	SCN Control	–	–	–	+
	5	Fluopyram + SCN	Fluopyram	0.15	mg ai/seed	+
	6	Pydiflumetofen + SCN	Pydiflumetofen	0.075	mg ai/seed	+

Tri-Cornered polypropylene beakers (Fisher Scientific - Hanover Park, IL, USA) with 400mL capacity were used as planting pots. Pots were filled with a 2:1 sand: soil mix previously steam-pasteurized. Pots were placed in a water bath system with temperature control, as described by Hashmi et al. (2005). Greenhouse conditions were set to a 14h light cycle, the daytime temperature of 30°C, the nighttime temperature of 22°C, and the soil temperature (water bath) of 25°C. SCN eggs used for inoculation were from an HG type 2 (race 5) isolate collected in Randolph County, IL. Seedlings were infected with 2000 eggs extracted from a greenhouse increase of the nematode. Seeds were planted to a depth of 2.54 cm, and the inoculum was pipetted over seeds, which were then covered with the sand: soil mix. Plants were watered twice daily, keeping moisture close to water holding capacity.

### Plant growth evaluations

Seedling emergence (EM) was recorded daily, whereas plant height (PH – cm), stem diameter (SD – mm), and chlorophyll readings were collected at both 14 and 28 days after planting. Chlorophyll levels (µmol m^-2^) were estimated using a SPAD 502 Plus Chlorophyll Meter (Spectrum Technologies – Aurora, IL, USA). Phytotoxicity was assessed 7 and 10 DAP using a 0-10 scale, where 1 indicates 0-10% of cotyledon covered with phytotoxicity damage, …, and 10 denotes 90-100% of cotyledons covered with phytotoxicity symptoms.

### Root scanning and SCN counts

SCN cysts were collected from treatments inoculated with SCN by washing roots and wet-sieving through nested 707-μm-pore and 250-μm-pore sieves (Niblack et al., 2009). Eggs were extracted from cysts using the protocol described by Faghihi and Ferris (2000). Eggs were stained with acid fuchsin and observed using a Nikon SMZ-645 stereoscope. SCN population densities were expressed in eggs/cm^3^ of soil.

Following cyst extraction, roots from all treatments, with and without SCN, were weighed (RW - g) and scanned using a flat-bed scanner (Model Perfection V850; Epson - Los Alamitos, CA, USA). Images were collected at 600 dpi and analyzed using WinRHIZO 2020 (Regent Instruments - Canada, Quebec, Canada). Debris was filtered by removing objects with an area <0.0010 cm^2^ (Cousins and Murren, 2017). The following root parameters were estimated: total root length (RL - cm), surface area (SA - cm²), projected area (PA - cm²), total volume (TV - cm³), and average diameter (AD - cm).

### RNA extraction and sequencing

Roots were collected at 5 and 10 DAI from the seedling without SCN inoculation for RNA extraction. Roots were washed with tap water, dried with paper towels, placed in Falcon^®^ tubes (Fisher Scientific - Hanover Park, IL, USA), and immediately stored in a liquid nitrogen cooler. RNA was extracted using an RNeasy Mini Kit (QIAGEN – Germantown, MD, USA), with a step to remove DNA contamination using DNase I. RNA quality was checked in a bleaching gel (Aranda et al., 2012) and initially quantified using a Nanophotometer^®^ P-Class (Implen Inc, Westlake Village, CA, USA). Subsequently, final RNA concentrations, 28S/18S and 23S/16S ratios, and RIN/RQN scores were determined using an Agilent 2100 Bioanalyzer™ (Agilent Technologies, Santa Clara, CA, USA) (**Table S1**).

Library preparation and sequencing were performed by BGI Genomics in a DNBSEQ-T7 platform (Shenzhen, Guangdong, China), with an output of 35 million clean pair-end reads or 7 GB of clean data per sequenced sample.

### Data processing and analysis

After sequencing, raw reads were filtered, including steps to remove adaptor sequences, contamination, and low-quality reads using SOAPnuke. Reads were deleted when having *i*) more than 40% bases having a quality value lower than 20; *ii*) more than 3% of N in the read; *iii*) more than 28% of a sequence matching the adapter sequence. **Table S1** shows statistics of the dataset after cleaning steps were performed.

Clean reads were aligned to the soybean genome from cultivar Williams82 *Wm82.a4.v1* (Valliyodan et al., 2019) using STAR 2.7 (Dobin et al., 2013) running in the galaxy server (*usegalaxy.org*) (Afgan et al., 2018). A count matrix was downloaded for further analysis.

### Differentially expressed genes and gene ontology analysis

A set of bioinformatics tools were used to identify transcriptomic shifts and metabolic pathways in soybean after seed treatment with SDHI fungicides. Differentially expressed genes (DEG) were normalized against their respective controls (mock 5 DAP or mock 10 DAP) and identified using DESeq2 (Love et al., 2014). For each time point and treatment, pairwise comparisons were executed comparing treatments to mock control, using all four replicated libraries sequenced for each treatment. DESeq2 output includes fold changes (logFC) and adjusted p-values. P-values were adjusted using the Benjamini and Hochberg (1995) method. DEGs across treatments were identified by filtering DESeq2 results using an adjusted P-value <0.05, regardless of logFC. Uniquely expressed genes across treatments were explored using Venn diagrams using the interactive tool (Heberle et al., 2015).

The gene ontology (GO) system (Ashburner et al., 2000) was used to classify functions of DEGs filtered by the criteria above. Analyzes were performed using AgriGO v2.0 (Tian et al., 2017), using Fisher Test as the statistical method, and Benjamini and Yekutieli (2001) as the multi-test adjustment (FDR<0.05). Finally, a KEGG pathway analysis was conducted using ShinyGO (Ge et al., 2020) with an FDR<0.05 cutoff.

### SCN penetration

A final independent greenhouse assay was conducted, with six replicates, to assess SCN root penetration in soybean seedlings treated with fluopyram and pydiflumetofen. Seedlings were treated following rates included in **Table 1** and inoculated with 2000 SCN eggs at planting. All growth conditions were previously described. Shortly after infection, at 10 DAP, roots were collected, washed and the number of nematodes in each root was estimated using the acid fuchsin method (Byrd et al., 1983). To standardize SCN penetration data, the number of nematodes per root system was converted to the number of nematodes per gram of root.

### Data availability

Sequence datasets were submitted to the NCBI (National Center for Biotechnology Information) Sequence Read Archive (SRA) and are available under the accession number PRJNA781213.

## Results

### Plant growth and root parameters

A greenhouse trial was conducted to investigate the potential effects of seed treatments and SCN inoculation on plant growth and root parameters. Seed treatments had a significant influence on plant phytotoxicity at 7 DAP (P<0.0001), phytotoxicity at 10 DAP (P<0.0001), plant height at 14 DAP (P<0.0001), plant height at 28 DAP (P=0.0003), stem diameter at 14 DAP (P=0.0107), root weight (P=0.0020) and root surface area (P=0.0271) (**Table 2**).

**Table 2:** ANOVA (Analysis of variance) table indicating the effect of seed treatments on plant growth, root parameters, and SCN counts.

Variable/Statistics	Timing (DAP)	DF	Sum of Squares	F Ratio	Prob > F
Emergence (DAP)	1-7	5	1.5750751	1.0044	0.4316
Phytotoxicity	7	5	159.50751	11.4697	<.0001*
Phytotoxicity	10	5	287.50751	27.7559	<.0001*
Plant Height (cm)	14	5	29.854306	8.0294	<.0001*
Plant Height (cm)	28	5	50.914214	6.7594	0.0003*
Stem Diameter (cm)	14	5	1.5592097	3.648	0.0107*
Stem Diameter (cm)	28	5	4.9399843	1.5747	0.1985
Chlorophyl (µmol m^-2^	14	5	63.051889	0.7855	0.5683
Chlorophyl (µmol m^-2^)	28	5	147.32975	1.3699	0.2655
Root Weight (g)	30	5	71.136262	4.8999	0.0020*
Total Root Length (cm)	30	5	7275625.9	0.7773	0.5736
Root Surface Area (cm^2^)	30	5	28315.457	2.953	0.0271*
Root Projected Area (cm^2^)	30	5	72906.14	1.4584	0.2317
Root Volume (cm^3^)	30	5	224.3525	1.9252	0.1185
Root Average Diameter (mm)	30	5	5.3305944	0.9304	0.4753
SCN Cysts	30	2	8.110876	10.7862	0.0017*
SCN Eggs	30	2	14.015919	10.5453	0.0019*
SCN Fecundity (eggs per cyst)	30	2	0.9752688	0.9899	0.3980

*Bold denotes significant difference (P<0.05).

Only fluopyram-treated plants, with or without SCN inoculation, displayed phytotoxicity symptoms at 7 and 10 DAP (**Table 3**). Phytotoxicity symptoms were limited to soybean cotyledons and disappeared as plants developed. Plant height was lower at 14 DAP in fluopyram-treated plants than in mock and SCN control. In comparison, fluopyram + SCN plants had lower plant height than mock SCN control pydiflumetofen and pydiflumetofen + SCN (**Table 3**). At 28 DAP, fluopyram-treated plants, with or without SCN, averaged lower plant height than all additional treatments. The total root weight at the end of the experiment in fluopyram + SCN was lower than mock, pydiflumetofen, and pydiflumetofen + SCN (**Table 3**).

**Table 3:** Effect of seed treatments on plant emergence, phytotoxicity, growth parameters, and root measurements.

Treatment/Variable	Emergence			Phytotox. 7 DAP			Phytotox. 10 DAP			Height 14 DAP			Height 28 DAP			
	DAP			1-10			1-10			cm			cm			
	Mean	SE	Group^1^	Mean	SE	Group	Mean	SE	Group	Mean	SE	Group	Mean	SE	Group
1-Mock	3.00	0.23	a	0.00	0.00	b	0.00	0.00	b	9.18	0.35	a	12.42	0.55	a
2-SCN Control	3.67	0.32	a	0.00	0.00	b	0.00	0.00	b	9.67	0.50	a	12.77	0.71	a
3-Fluopyram	3.44	0.19	a	3.89	0.56	a	5.44	0.48	a	7.66	0.30	bc	10.09	0.43	b
4-Fluopyram + SCN	3.33	0.23	a	4.67	0.68	a	6.00	0.59	a	6.65	0.35	c	10.07	0.50	b
5-Pydiflumetofen	3.25	0.20	a	0.00	0.00	b	0.00	0.00	b	8.30	0.30	ab	12.26	0.43	a
6-Pydiflum. + SCN	3.00	0.25	a	0.00	0.75	b	0.00	0.00	b	8.60	0.39	ab	12.84	0.55	a
**Treatment/Variable**	SD 14 DAP			SD 28 DAP			Chlorop. 14 DAP			Chlorop. 28 DAP			Root Weight			
	cm			cm			µmol m^-2^			µmol m^-2^			g			
	Mean	SE	Group	Mean	SE	Group	Mean	SE	Group	Mean	SE	Group	Mean	SE	Group
1-Mock	2.41	0.12	ab	3.17	0.35	a	43.53	1.64	a	32.54	2.07	a	10.73	0.70	a
2-SCN Control	1.93	0.17	b	2.77	0.46	a	40.20	2.31	a	31.27	2.68	a	7.60	0.98	ab
3-Fluopyram	2.34	0.10	ab	3.14	0.28	a	42.35	1.42	a	31.46	1.75	a	8.74	0.57	ab
4-Fluopyram + SCN	2.75	0.12	a	3.99	0.32	a	41.63	1.64	a	32.58	1.89	a	6.15	0.70	b
5-Pydiflumetofen	2.29	0.10	ab	2.94	0.28	a	40.63	1.42	a	27.99	1.64	a	9.01	0.60	a
6-Pydiflum. + SCN	2.51	0.13	ab	3.38	0.35	a	39.44	1.79	a	34.20	2.07	a	9.46	0.76	a
**Treatment/Variable**	Total Root Length			Root Surface Area			Projected Area			Root Volume			Average Diameter			
	cm			cm^2^			cm^2^			cm^3^			mm			
	Mean	SE	Group	Mean	SE	Group	Mean	SE	Group	Mean	SE	Group	Mean	SE	Group
1-Mock	891.29	558.58	a	433.02	17.88	a	236.81	40.82	a	16.36	1.97	a	1.80	0.44	a
2-SCN Control	1021.30	789.95	a	394.73	25.28	abc	125.65	57.73	a	12.50	2.79	a	1.26	0.62	a
3-Fluopyram	1133.75	456.08	a	390.61	14.60	abc	124.34	33.33	a	11.14	1.61	a	1.15	0.36	a
4-Fluopyram + SCN	1183.39	558.58	a	347.15	17.88	c	110.50	40.82	a	8.41	1.97	a	0.99	0.44	a
5-Pydiflumetofen	2098.56	483.75	a	366.70	15.48	bc	116.74	35.35	a	10.39	1.71	a	1.99	0.38	a
6-Pydiflum. + SCN	945.35	611.90	a	409.54	19.58	ab	109.38	44.72	a	13.19	2.16	a	1.30	0.54	a

^1^Means followed by same letters are not statistically different using Tukey’s HSD (P=0.05). All root measurements were conducted at 30 DAP.

### SCN reproduction

SCN-inoculated treatments were evaluated to assess the impact of seed treatment on nematode reproduction. Seed treatment had a significant effect on both SCN cysts (P=0.0017) and egg counts (P=0.0019) but not on cyst fecundity (P=0.3980) (**Table 4**). Fluopyram reduced both SCN egg and cyst counts compared to pydiflumetofen and control. On average, cyst counts in SCN control and pydiflumetofen treated plants were 3.44 and 3.59 times higher than fluopyram, respectively, while egg counts were 8.25 and 7.06 times higher in SCN control and pydiflumetofen (**Table 4**). A trend of lower SCN fecundity in fluopyram was observed, although it was not statistically significant.

**Table 4:** Effect of treatments on the number of cysts, eggs, and egg fecundity (eggs per cyst).

Treatment/Variable	SCN Cysts			SCN Eggs			SCN Fecundity		
	Mean	SE	Group^1^	Mean	SE	Group^1^	Mean	SE	Group^1^
2 - SCN Control	25.25	2.84	A	990.00	254.41	A	40.0296	9.46	A
4 - Fluopyram + SCN	7.33	2.32	B	120.00	207.73	B	19.2723	7.73	A
6 - Pydiflumetofen + SCN	26.33	2.32	A	846.67	207.73	A	31.2275	7.73	A

^1^Means followed by the same letter in the column are not statistically different using Tukey’s HSD test (P=0.05). SCN count data was log-transformed to follow the normal distribution.

### SCN penetration

In an independent greenhouse study, seed treatment significantly affected SCN penetration (P=0.0112). The number of SCN juveniles per gram of root was reduced considerably in fluopyram-treated plants (-82.10%). A trend of a lower number of juveniles was also observed in pydiflumetofen (-19.56%), but the results were comparable to the control. All results are summarized in **Table 5**.

**Table 5:** Number of SCN juveniles per gram of root following seed treatment with fluopyram and pydiflumetofen (P=0.0112).

Treatment/Variable	SCN Cysts		
	Mean	SE^1^	Group^2^
SCN Control	254.54	44.02	A
Fluopyram	45.56	44.02	B
Pydiflumetofen	204.75	44.02	A

^1^SE was calculated using a pooled estimate of error variance. ^2^Means followed by the same letter in the column are not statistically different using Tukey’s HSD test (P=0.05). SCN count data was log-transformed to follow the normal distribution.

### Soybean transcriptomic profiles by fluopyram and pydiflumetofen

A greenhouse trial was conducted to assess gene expression patterns in soybean plants treated with fluopyram and pydiflumetofen. Samples were collected from treatments (mock, fluopyram, and pydiflumetofen) at two different time points (5 and 10 DAP). Each sampling included four biological replicates per treatment, sequenced to a depth of 35 million reads per sample. An average of 93.45% ( ± 0.92) of reads were uniquely aligned to the soybean genome, while 1.86% ( ± 0.11) aligned to multiple loci and 4.67% ( ± 0.83) were not aligned (**Table S2**). Only uniquely aligned reads were used for downstream analysis.

**Figure 1:**
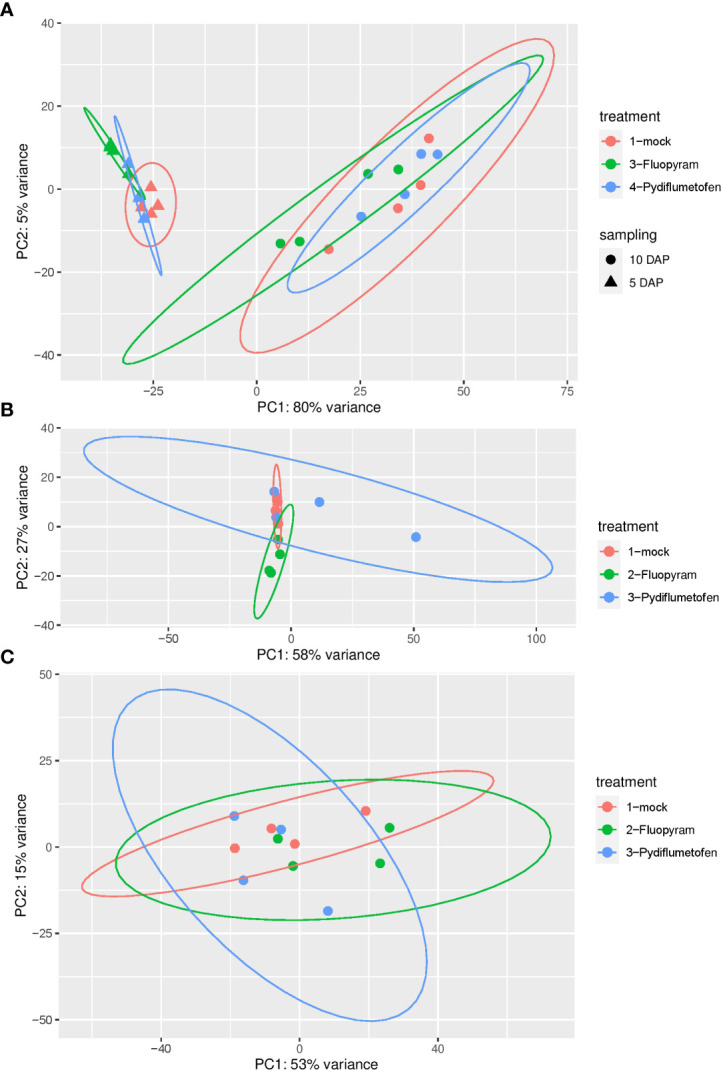
Principal coordinate analysis (PCA) of gene expression in soybean seedlings treated with fluopyram and pydiflumetofen 5 and 10 days after planting **(A)**. PCA analysis was also conducted for each time point (**B**: 5 DAP; **C**: 10 DAP).

After further analysis, expression patterns of treatments clustered by sampling time (5 DAP vs. 10 DAP), as shown by the principal coordinate (PCA) analysis (**Figure 1A**). PCA analysis was also conducted for each time point (**Figure 1B**: 5 DAP; **Figure 1C**: 10 DAP). Since expression profiles were strongly affected by sampling time, treatments were separated by each sampling for downstream analyses.

**Figure 2:**
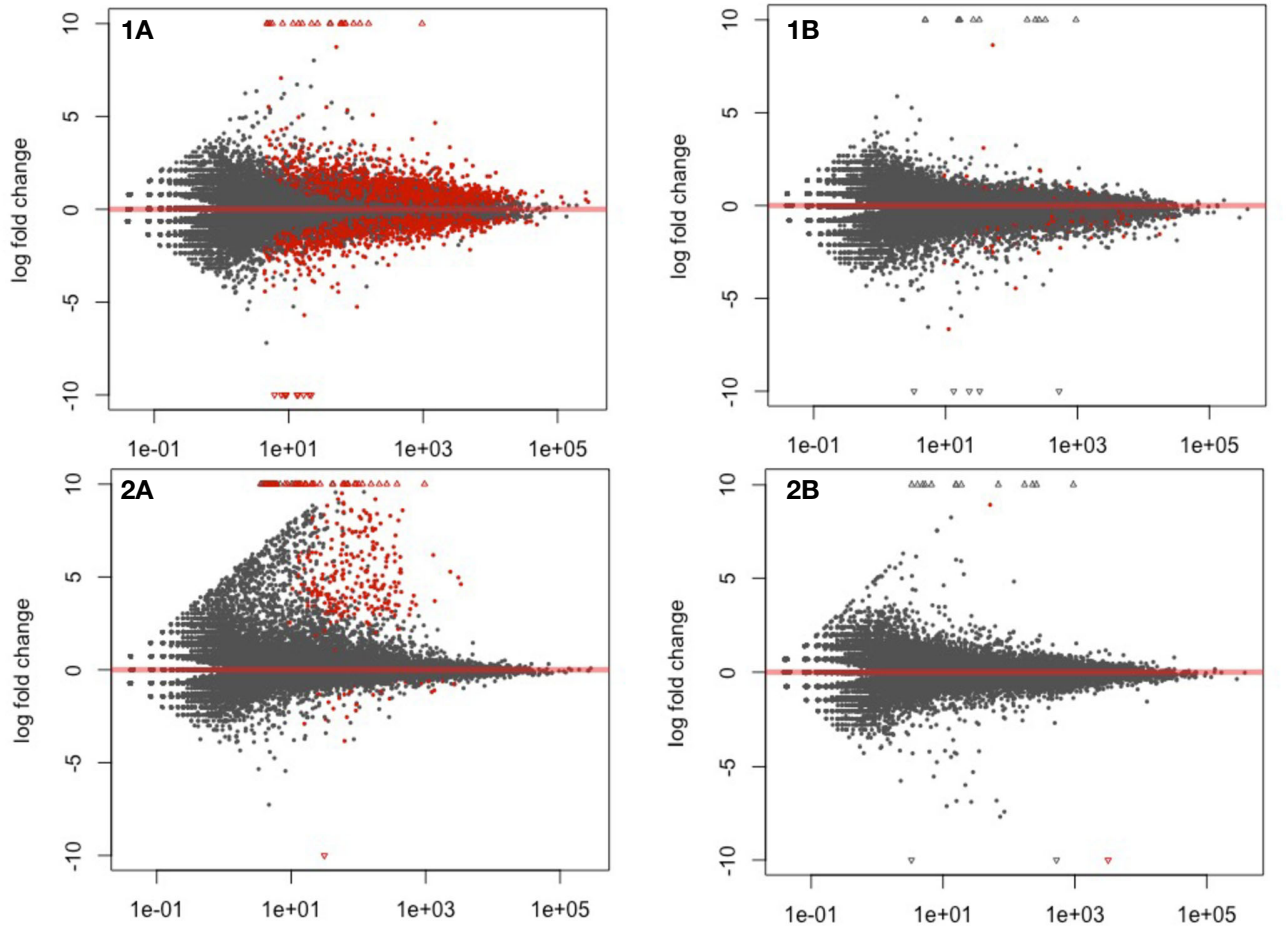
Volcano plot highlighting differentially expressed genes in soybean seedlings treated with fluopyram and pydiflumetofen, 5 and 10 days after planting (DAP). **1A**: Fluopyram 5 DAP; **1B**: Fluopyram 10 DAP; **2A**: Pydiflumetofen 5 DAP; **2B**: Pydiflumetofen 10 DAP. Red dots represent differentially expressed genes (DEGs) (Padj<0.05).

To standardize gene expression, the treatments (fluopyram and pydiflumetofen) were normalized against their respective controls (mock 5 DAP or mock 10 DAP). A series of volcano plots highlight all differentially expressed genes (P<0.05) in **Figure 2**. A sharp decline in the number of differentially expressed genes was detected when comparing the same treatment across timepoints (5 DAP versus 10 DAP).

**Figure 3:**
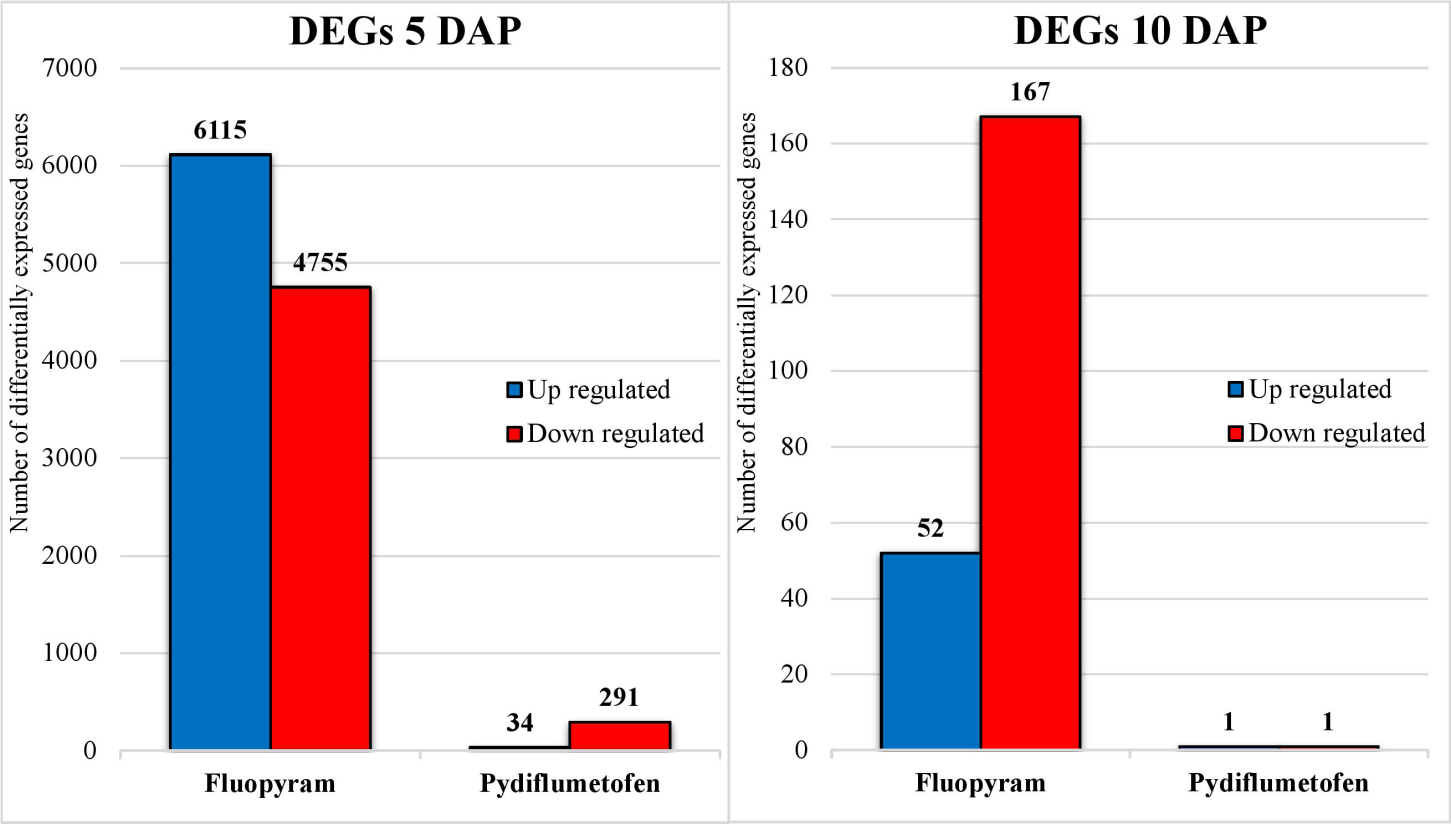
Number of differentially expressed genes (DEGs) up and downregulated in soybean seedlings treated with fluopyram and pydiflumetofen, 5 and 10 days after planting (DAP). All gene expression levels were compared to their respective non-treated control (5DAP or 10 DAP). Genes were considered differentially expressed when having Padj<0.05, regardless of fold change (logFC).

The parenthesis percentage indicates the ratio of 50,424 soybean genes detected during analysis. At 5 DAP, using a cutoff of Padj<0.05, a total of 10,870 DEG were detected in fluopyram (21.56% - 6115 up, 4755 down) and 325 in pydiflumetofen (0.64% - 34 up, 291 down). At 10 DAP, 219 DEGs were identified in fluopyram (0.43% - 52 up, 167 down), and 2 in pydiflumetofen (0.004% - 1 up, 1 down). All results are summarized in **Figure 3**.

**Figure 4:**
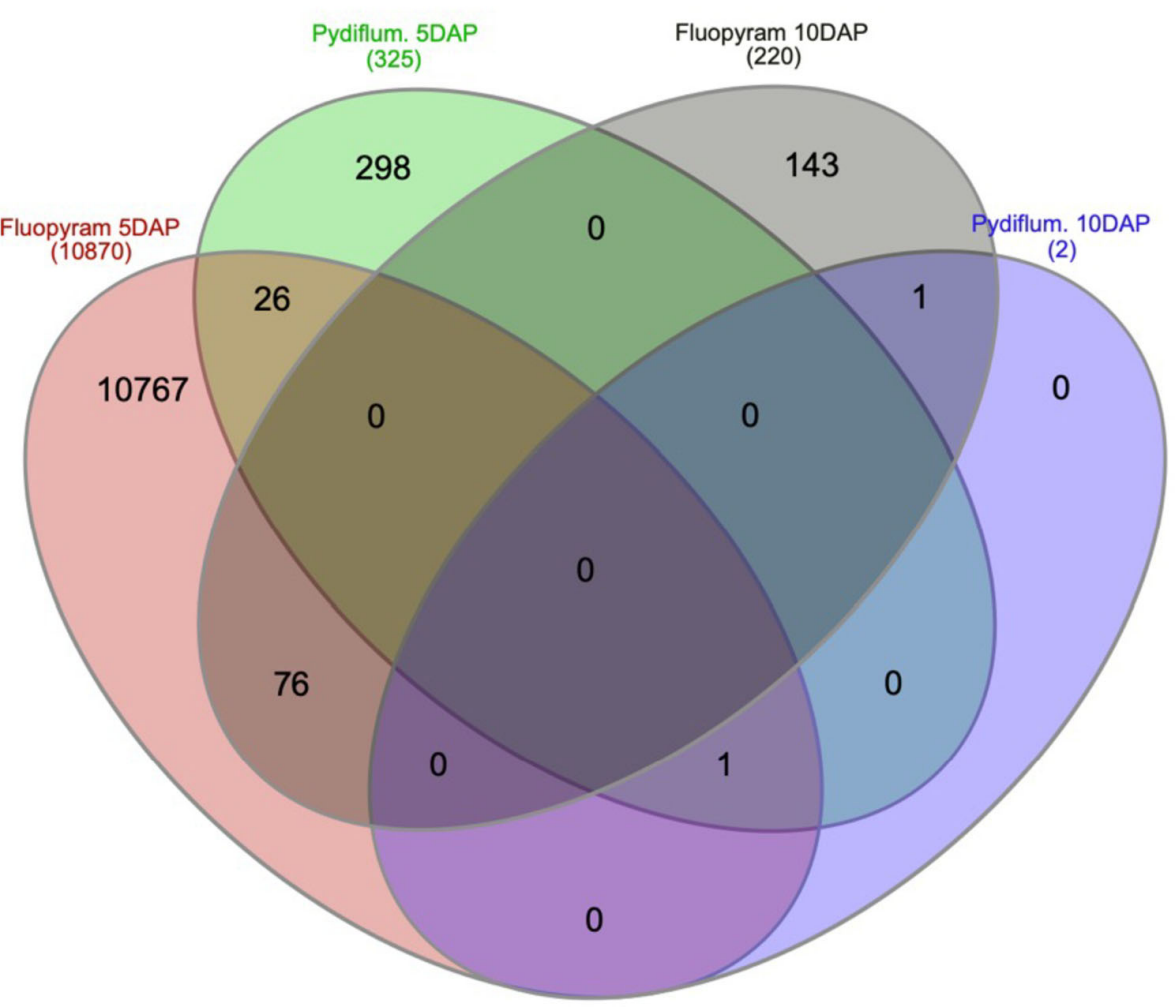
A Venn diagram plotting filtered DEGs with P-value<0.05 up and downregulated in soybean seedlings treated with fluopyram and pydiflumetofen, 5 and 10 days after planting (DAP). The number of DEGs shared between treatments is displayed by overlapping circles. All treatments were normalized against their respective controls (mock 5 DAP or mock 10 DAP).

A Venn diagram was plotted to display unique DEGs across treatments and time points (**Figure 4**). As shown in **Figure 4**, a sum of 10767, 298, 143, and 0 genes were uniquely expressed in fluopyram 5 DAP, pydiflumetofen 5 DAP, fluopyram 10 DAP, and pydiflumetofen 10 DAP, respectively. Fluopyram 5 DAP and fluopyram 10 DAP shared 76 DEGs, while fluopyram 5 DAP and pydiflumetofen 5 DAP had 26 common DEGs.

### Systemic resistance-related genes in soybean treated with fluopyram and pydiflumetofen

The effect of fluopyram and pydiflumetofen on a list of genes linked to soybean systemic resistance is summarized in **Table S4**. Out of 54 genes linked to systemic resistance in soybean summarized from DESeq2 data, 26 and 1 DEGs with a Padj<0.05 were identified for fluopyram 5 DAP and pydiflumetofen 5 DAP. From this list, *Glyma.13G106400* (LogFC: 5.50), *Glyma.13G234400* (LogFC: 4.48), *Glyma.01G130500* (LogFC: 2.88), *Glyma.19G229500* (LogFC: 2.64) and *Glyma.07G20990* (LogFC: 2.11) had the highest LogFC in fluopyram 5 DAP. In fluopyram 10 DAP, only 2 genes were identified, both downregulated: *Glyma.03G038100* (logFC: -1.42) *Glyma.03G247500* (LogFC: -2.52), perhaps a result of gene expression autoregulation. *Glyma.02G209000* (LogFC: 4.02) was the only gene upregulated in pydiflumetofen at 5 DAP. **Table S4** also lists all genes involved in both jasmonic and salicylic acid pathways using data from SoyBase SoyCyc 10.0 (Grant et al., 2010). A list of key genes was significantly upregulated in Fluopyram 5 DAP, as examples of *Glyma.15G067800* (PR1), *Glyma.09G020800* (NPR1/NIM1 like defense protein C terminal), *Glyma.03G181700* (PAL 1.2), *Glyma.10G0582200* (PAL 2.1) and *Glyma.19G182300* (PAL 1.1).

**Figure 5:**
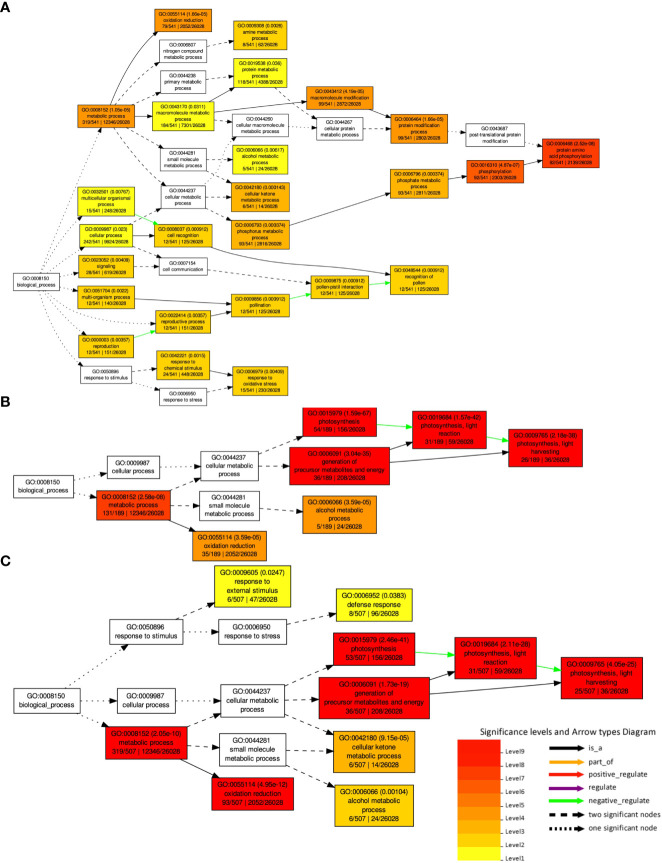
Gene ontology (GO) analysis and hierarchical classification of deferentially expressed genes DEGs by fluopyram at 5 DAP **(A)**, pydiflumetofen 5 DAP **(B)** and fluopyram 10 DAP **(C)**. No significant GO terms were identified in pydiflumetofen at 10 DAP. Only upregulated genes with logFC >1.0 were used in this analysis.

**Figure 6:**
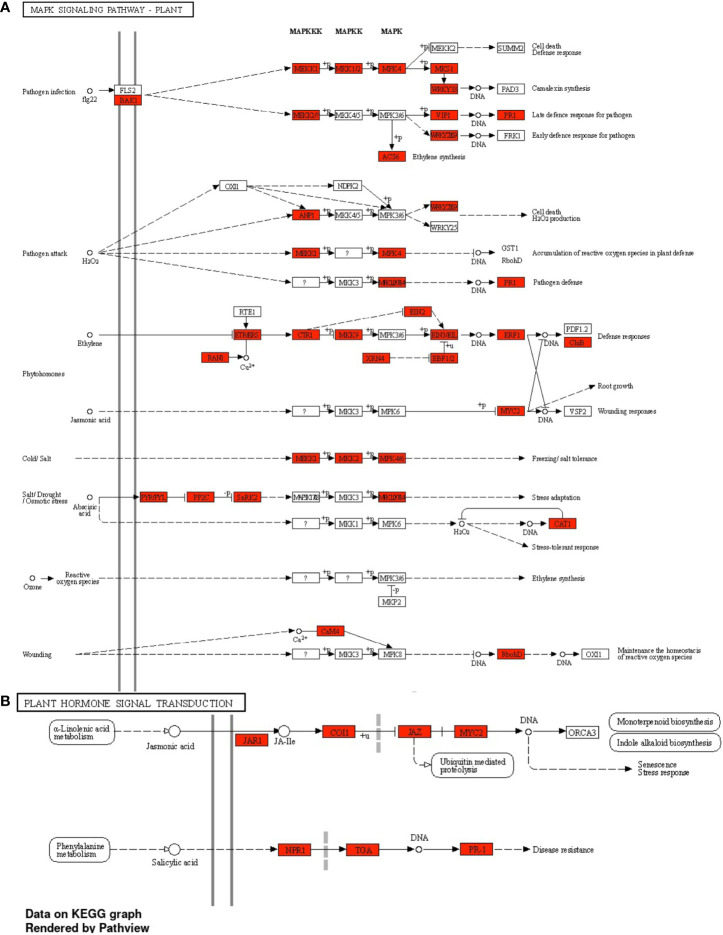
MAPK signaling **(A)** and plant hormone signal transduction **(B)** pathways in response to fluopyram in soybean seedling 5 days after planting. KEGG pathway analysis was conducted using ShinyGO (Ge et al., 2020) with a FDR<0.05 cutoff.

**Figure 7:**
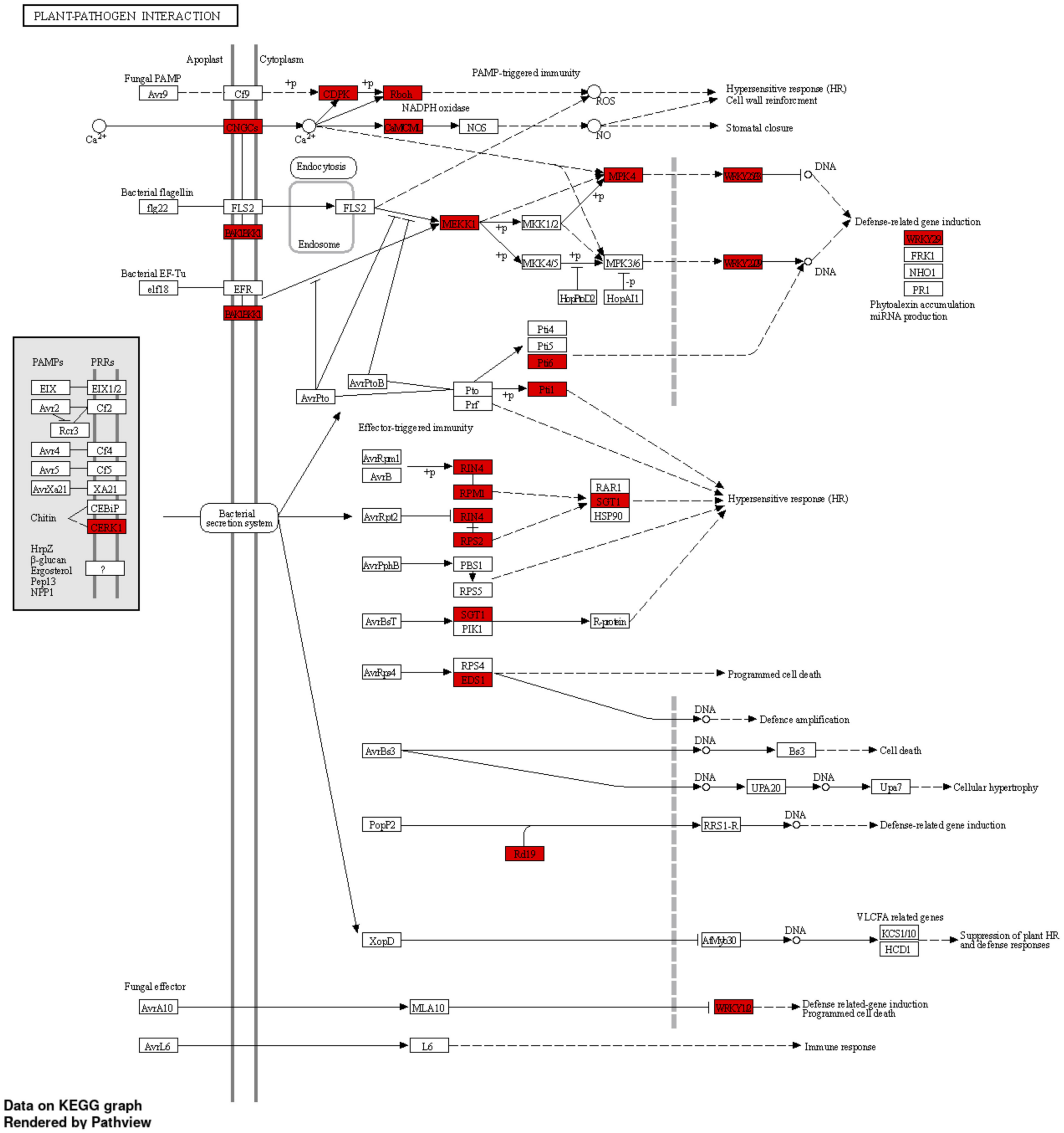
Plant-pathogen interaction pathways in response to fluopyram in soybean seedling 5 days after planting. KEGG pathway analysis was conducted using ShinyGO (Ge et al., 2020) with a FDR<0.05 cutoff.

A GO enrichment analysis was performed for the soybean upregulated genes at 5 DAP (**Figure 5**). Like results from **Table S5**, where multiple soybean genes associated with systemic resistance were significantly upregulated in fluopyram-treated plants, the GO enrichment analysis indicates several GO terms linked to systemic resistance being activated with fluopyram. These terms were only identified in fluopyram-treated plants and include response to stimulus (GO:0050896), response to external stimulus (GO:0009605), response to stress (GO:0006950), and defense response (GO:0006952). Furthermore, a KEGG pathway analysis was conducted for fluopyram 5 DAP, revealing a series of genes significantly expressed within the MAPK (mitogen-activated protein kinases) signaling (**Figure 6**) and plant-pathogen interaction (**Figure 7**) pathways, including PR1-related pathways. Both jasmonic and salicylic acid pathways were stimulated by fluopyram.

From a list of 121 genes related to succinate dehydrogenase and/or ubiquinone obtained from the SoyBase SoyCyc 10.0 (Grant et al., 2010), 50 genes were significantly expressed across all treatments and time points (**Table S5**). Most of these genes were only expressed at 5 DAP in fluopyram-treated plants (50 out of 51). *Glyma.05G139800* was the only upregulated gene in pydiflumetofen across both time points. At 10 DAP, only one ubiquinone-related gene was detected across all treatments, the gene *Glyma.06G298300* (ubiquinone oxidoreductase) was downregulated in seedlings treated with fluopyram (LogFC: -4.45; P=0.048675).

## Discussion

A set of greenhouse assays were conducted to evaluate the effect of two SDHI (succinate dehydrogenase inhibitors) seed treatments (fluopyram and pydiflumetofen) on soybean development and SCN reproduction. Next-generation sequencing technologies were also employed to identify potential transcriptomic shifts in gene expression profiles of soybean seedlings treated with fluopyram and pydiflumetofen. In the present research, we investigate if the suppression of SCN by these two chemicals could be linked to the activation of plant systemic resistance.

Fluopyram-treated plants displayed early phytotoxicity symptoms, which were limited to the cotyledons and disappeared as plants developed. Seedlings treated with fluopyram consistently show phytotoxicity often referred to as the “halo effect” (Kandel et al., 2018). This early phytotoxicity may also influence early plant development since plant height was similarly affected in these plants at both 14 and 28 DAP. We further analyzed how fluopyram and pydiflumetofen affected SCN root penetration, reproduction, and final population densities. Fluopyram significantly reduced SCN root penetration cyst and egg counts compared to pydiflumetofen and control. The number of SCN eggs per cyst, or fecundity, was similar across treatments. Our results corroborate previous studies in the literature, which reported fluopyram reducing SCN mobility and root penetration rates (Kandel et al., 2017; Beeman and Tylka, 2018; Beeman et al., 2019; Roth et al., 2020). Both fluopyram and pydiflumetofen are included in commercial seed treatments recommended for managing SCN and soybean sudden death syndrome (SDS) caused by *Fusarium virguliforme*. As previously discussed, the broad range of activity of the compounds, especially fluopyram, raised a question about the potential activation of systemic resistance.

The gene expression profiles of soybean seedlings treated with fluopyram and pydiflumetofen were analyzed using next-generation sequence technologies. Gene expression patterns clustered by sampling time (5 DAP vs. 10 DAP); therefore, downstream analyses were conducted, separating treatment by each sampling. At 5 DAP, fluopyram strongly influenced soybean gene expression patterns compared to pydiflumetofen. The sharp contrast between fluopyram and control plants may be partly related to the delayed development in fluopyram-treated plants, as seedlings in different developmental stages display contrasting expression patterns. The number of DEGs in fluopyram-treated plants significantly dropped at 10 DAP. Although pydiflumetofen had a minor early influence on gene expression profiles, the later sampling (10 DAP) revealed that expression patterns were comparable to the control. After further analysis using the GO classification system, multiple soybean genes significantly upregulated in fluopyram-treated plants were linked to systemic resistance. To our knowledge, this is the first report of systemic resistance in soybean activated by fluopyram.

Another significant discovery in our study was the first report of the downregulation of succinate dehydrogenase (SDH) and ubiquinone oxidoreductase genes in soybean by fluopyram. Multiple genes involved in these metabolic routes were significantly downregulated. The mechanism of action in SDHI fungicides, including fluopyram, relies on inhibiting succinate dehydrogenase within the fungal mitochondrial respiration chain by binding to ubiquinone-binding sites (Qp) in the succinate dehydrogenase complex (Sang et al., 2018). Since SDH in an enzyme complex shared across eukaryotes, this compound can theoretically bind to the same target in soybean, which was supported by our study. The phytotoxicity frequently observed in soybean seedlings is thought to be linked to the metabolism of fluopyram and its breakdown of metabolites within the soybean plant (Robatscher et al., 2019). Still, those mechanisms were yet to be clarified. In *Arabidopsis thaliana* and *Oryza sativa*, SDH was demonstrated to be a direct source of reactive oxygen species (ROS) in plant mitochondria, accompanied by the down-regulation of cell cycle genes and the up-regulation of stress-related genes. The induction of ROS production by specific SDH inhibitors regulates plant growth and increases stress-related genes (Jardim-Messeder et al., 2015). SDH-dependent ROS production was proposed as an additional mechanism to explain pathogen resistance and oxidative damage in plant cells (Huang and Millar, 2013; Huang et al., 2019). The non-target inhibition of soybean SDH genes by fluopyram may be the origin of the phytotoxicity symptoms observed and potentially cause the systemic resistance activation reported in the current study.

One key aspect to consider when looking at early seed treatments, chemical or biological-based, is systemic resistance priming. Plants are induced to a sensitized state, responding more efficiently to subsequent attacks by pathogens (Shoresh et al., 2005). This early stimulation of resistance pathways by fluopyram may enable plants to become more resistant to later pathogen infection. Although the early activation of systemic resistance genes by fluopyram may not be observable in later soybean growth stages, the priming effect may allow the plant to respond to later pathogen infections promptly. Several examples are described in the literature, mainly focusing on priming resistance by beneficial microorganisms. Plant-associated bacteria, including *Pseudomonas* sp., were shown to induce priming of resistance-related phenolic compounds, resulting in reduced infections of *Hymenoscyphus fraxineusin* in European ash trees (*Fraxinus excelsior*) (Striganavičiūtė et al., 2021). Cucumber plants pre-inoculated with *Trichoderma* spp. had a higher systemic expression of pathogenesis-related genes in a later infection with the leaf pathogen *Pseudomonas syringae* pv. *lachrymans* (Shoresh et al., 2005) . *Trichoderma hamatum* similarly primed *Arabidopsis thaliana*, resulting in an accelerated activation of the defense response against *Botrytis cinerea* (Mathys et al., 2012). Soybean seedlings treated with oxo-C14-HSL had reduced reproduction of the root-lesion nematode, *Pratylenchus penetrans* (Adss et al., 2021). Noteworthily, priming was systemically induced, persisted after removing the priming agent (oxo-C14-HSL), and produced no defense markers before nematode attack.

In summary, fluopyram reduced both SCN egg and cyst counts compared to pydiflumetofen and untreated control. Fluopyram-treated plants also displayed lower plant height and phytotoxicity, the latter being a common symptom in plants treated with this compound. RNA expression patterns of seed treatments clustered by sampling time (5 DAP vs. 10 DAP). A sum of 10870, 370, 219, and 2 differentially expressed genes (DEG) were identified in fluopyram 5 DAP, pydiflumetofen 5 DAP, fluopyram, 10 DAP, and pydiflumetofen 10 DAP, respectively. Multiple DEGs identified in soybean seedlings treated with fluopyram are linked to systemic resistance, suggesting a potential role of systemic resistance in the suppression of SCN by fluopyram, in addition to the nematicidal activity of fluopyram. The non-target inhibition of soybean SDH genes by fluopyram may be the origin of the phytotoxicity symptoms observed and potentially the cause of the systemic resistance activation reported in the current study. This work confirms that fluopyram activates plant systemic resistance when used as a seed treatment, as initially suggested by Peng et al. (2020). To our knowledge, this is the first report of the suppression of soybean succinate dehydrogenase (SDH) and ubiquinone oxidoreductase genes by fluopyram. This research helps to elucidate the mechanisms of suppression of SCN by fluopyram.

## Data availability statement

Sequence datasets were submitted to the NCBI (National Center for Biotechnology Information) Sequence Read Archive (SRA) and are available under the accession number PRJNA781213.

## Author contributions

LR conducted sample processing, DNA extraction, and quality check with the assistance of AS and MF. LR analyzed the data and wrote the manuscript with help from AS, MF, AF, and JB. LR designed figures, formatted, and submitted the manuscript. All authors have read and agreed to the published version of the manuscript.

## Funding

This study received funding from BASF SE. The funder was not involved in the study design, collection, analysis, interpretation of data, the writing of this article or the decision to submit it for publication.

## Acknowledgments

This work is part of the first author’s Ph.D. dissertation. We are thankful for the assistance from SIU researchers, undergraduate and graduate students conducting trials, collecting samples, extracting RNA samples, and analyzing data. This project was completed using funding from BASF Corporation.

## Conflict of interest

This project was completed using funding from BASF Corporation. The authors declare no additional relationships that could be construed as a potential conflict of interest.

## Publisher’s note

All claims expressed in this article are solely those of the authors and do not necessarily represent those of their affiliated organizations, or those of the publisher, the editors and the reviewers. Any product that may be evaluated in this article, or claim that may be made by its manufacturer, is not guaranteed or endorsed by the publisher.
