# Supplementary figures and images for: Fluopyram activates systemic resistance in soybean
Source: Front Plant Sci. 2022 Oct 24;13:1020167. doi: 10.3389/fpls.2022.1020167 (PMC9638427; doi:10.3389/fpls.2022.1020167)

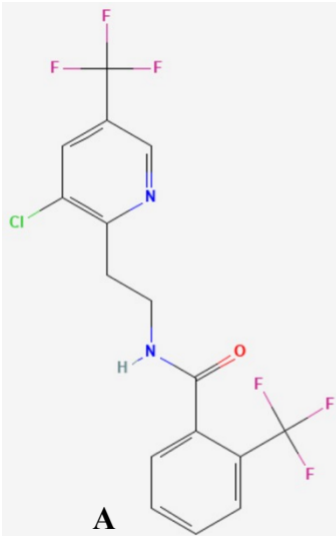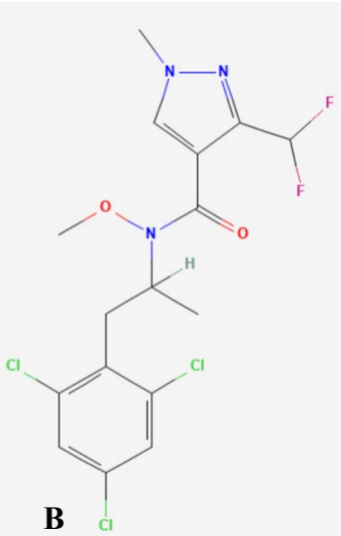

Supplement: Supplementary file 1 [file Image_1.pdf]
